# Prenatal Detection of Esophageal Atresia with Transposition of Great Arteries

**DOI:** 10.4274/balkanmedj.galenos.2019.2019.1.80

**Published:** 2019-05-10

**Authors:** Peng An, Weichao Liu, Yuxin Ning, Yingjian Ye, Yu Wang

**Affiliations:** 1 Department of Ultrasound, Xiangyang No. 1 People’s Hospital Affiliated to Hubei University of Medicine, Xiangyang Key Laboratory of Maternal-Fetal Medicine in Fetal Heart Disease, Hubei, China

A 31-year-old singleton pregnant woman (gravida 2, para 1) was admitted to the Maternal–Fetal Medical Center of the Xiangyang First People's Hospital Affiliated to Hubei Medical University on July 5, 2018, at 30 + 1 weeks of gestation, with a suspicion of fetal congenital heart disease. The nuchal translucency was 1.1 mm at 12 + 2 weeks of gestation. However, neither a noninvasive DNA analysis, genetic, nor karyotype assessment was performed. Prenatal echocardiography exhibited parallel initial segments of the aorta pulmonary arteries. The aorta originated from the right ventricle and the pulmonary artery from the left ventricle (Figure 1a). In addition, the fetal stomach was not seen (72 hour review); maximum amniotic fluid depth was 7.1 cm; and amniotic fluid index was 26.2 cm. Subsequently, the pregnant woman underwent a T2W1 magnetic resonance imaging examination, which showed an upper esophageal pouch (Figure 1b). The prenatal diagnostic conclusions were (1) evidence of transposition of the great arteries and (2) suspicion of type I esophageal atresia. Two days later, the pregnant woman was presented to the Shenzhen Maternal and Child Health Hospital for a re-examination and received the same diagnosis as above. Preliminary evaluation of the prenatal diagnostic centers and pediatric surgery department was fetal intrauterine development restriction, and the ultrasound gestational age (25 + 2 weeks) was significantly lower than the clinical gestational age (30 + 1 week). These results showed a high possibility of the fetus to have a birth weight less than 1.500 grams. The pregnant woman decided to terminate the pregnancy after considering the intricate modus operandi, possible complications after the surgery, and chances of a low survival rate. Magnetic resonance imaging of the specimen revealed a disconnected esophagus with a visible lower broken end. The anatomy also confirmed the presence of transposition of the great arteries (Figure 2a). After the thymus was separated, the upper end of the remaining esophagus had a length of 2.8 cm (Figure 2b, 2c). An acrylonitrile-butadiene-styrene polymer was injected through the umbilical vein by a 1.2-mm pediatric scalp needle, and appropriate amounts of red and yellow pigments were added as cast filler. After the specimen was filled and solidified, it was placed in a hydrochloric acid solution for corrosion. Finally, an ideal complete vascular cast was made, which revealed the transposition of the great arteries perfectly. Also, the cast could be permanently preserved and studied at any time (Figure 2d). Written informed consent was obtained from the mother.

Esophageal atresia combined with transposition of the great arteries is extremely rare, and it is extremely difficult to get an accurate prenatal diagnosis (1,2,3). Presently, esophageal atresia can only be diagnosed directly using prenatal ultrasound, which reveals an esophageal pouch that is remarkably difficult to locate. Therefore, esophageal atresia may be strongly suspected when polyhydramnios (amniotic fluid index ≥25) and a persistently absent stomach (72 hour review) are noted. However, due to the influence of maternal skin thickness, placenta, amniotic fluid, and tissues and organs around the esophagus, it is very difficult to detect the esophageal pouch solely based on ultrasound. Fortunately, an magnetic resonance imaging test conveys better results since it is not affected by the aforementioned factors. Ethun et al. (4) believed that with an appropriate scanning sequence, the magnetic resonance imaging can easily show the esophageal pouch, further confirming esophageal atresia. Therefore, subsequent magnetic resonance imaging is a very crucial approach when esophageal atresia is suspected.

In conclusion, prenatal ultrasound combined with magnetic resonance imaging can diagnose type I esophageal atresia and transposition of the great arteries and positively support the prenatal consultation. Casting technology can help in the real-time permanent preservation of rare deformities and can provide such materials for diagnostic knowledge sharing.

## Figures and Tables

**Figure 1a:**
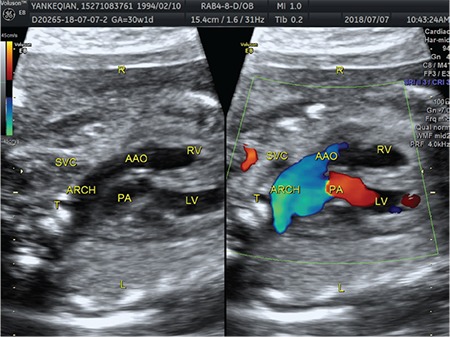
Prenatal ultrasound of a female, 30 + 1 weeks of gestation, showing TGA. Prenatal echocardiography exhibited parallel initial segments of the aorta Pas. AAO: ascending aorta; ARCH: aortic arch; LV: left ventricle; PA: pulmonary artery; RAA: right atrial appendage; RV: right ventricle; SVC: superior vena cava; T: trachea; TGA: transposition of the great arteries

**Figure 1b:**
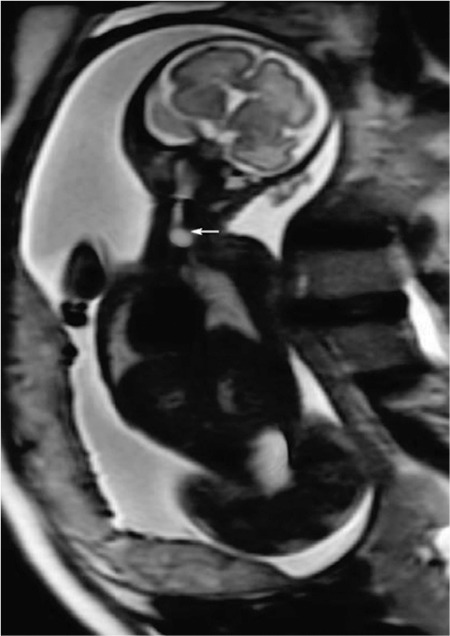
T2W1 magnetic resonance imaging shows the upper paraesophageal pouch.

**Figure 2a:**
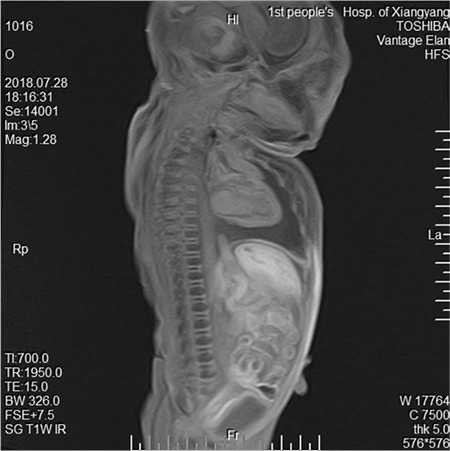
After inducing labor, magnetic resonance imaging of the specimen shows a disconnected esophagus with a visible lower broken end.

**Figure 2b:**
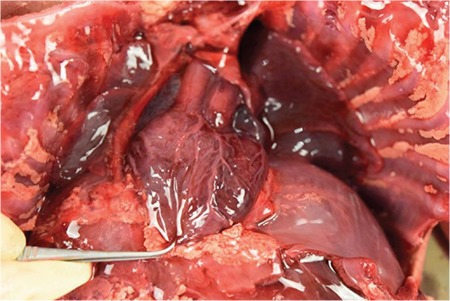
Autopsy reveals that the aorta and pulmonary artery were parallel in shape, confirming transposition of the great arteries.

**Figure 2c:**
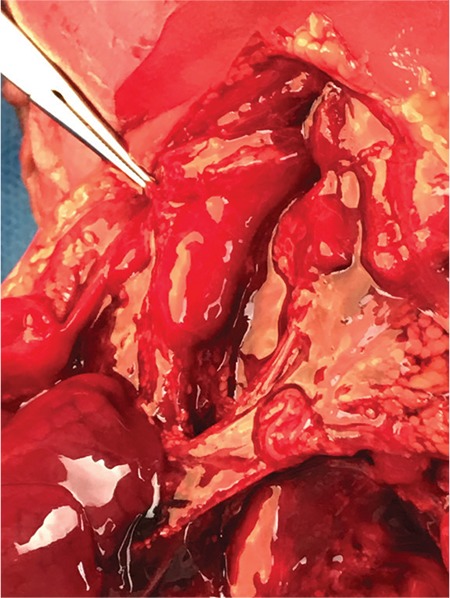
The upper esophagus was 2.8 cm long after the thymus was separated.

**Figure 2d:**
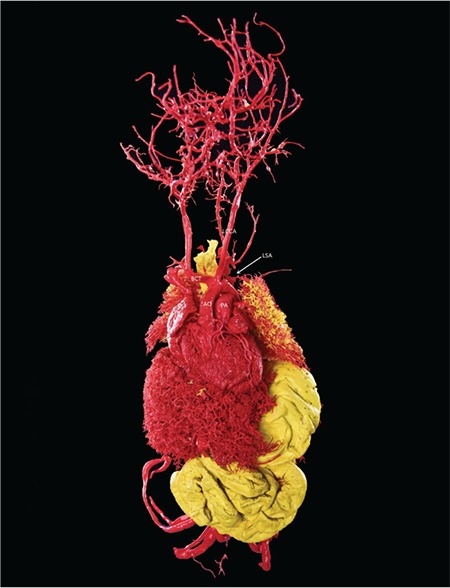
Vascular tracheal cast perfectly revealed an aberrant left subclavian artery (from the descending aorta) and the absence of ductus arteriosus. BCT: brachiocephalic trunk; LCCA: left common carotid artery; LSA: left subclavian artery; PA: pulmonary artery
